# New roles for the cerebellum in health and disease

**DOI:** 10.3389/fnsys.2013.00083

**Published:** 2013-11-14

**Authors:** Stacey L. Reeber, Tom S. Otis, Roy V. Sillitoe

**Affiliations:** ^1^Department of Pathology and Immunology, Department of Neuroscience, Baylor College of Medicine, Jan and Dan Duncan Neurological Research Institute of Texas Children's HospitalHouston, TX, USA; ^2^Department of Neurobiology, Geffen School of Medicine at University of California, Los AngelesLos Angeles, CA, USA

**Keywords:** neurological disorders, genetics, circuitry, neural activity, brain behavior

## Abstract

The cerebellum has a well-established role in maintaining motor coordination and studies of cerebellar learning suggest that it does this by recognizing neural patterns, which it uses to predict optimal movements. Serious damage to the cerebellum impairs this learning and results in a set of motor disturbances called ataxia. However, recent work implicates the cerebellum in cognition and emotion, and it has been argued that cerebellar dysfunction contributes to non-motor conditions such as autism spectrum disorders (ASD). Based on human and animal model studies, two major questions arise. *Does* the cerebellum contribute to non-motor as well as motor diseases, and if so, *how* does altering its function contribute to such diverse symptoms? The architecture and connectivity of cerebellar circuits may hold the answers to these questions. An emerging view is that cerebellar defects can trigger motor and non-motor neurological conditions by globally influencing brain function. Furthermore, during development cerebellar circuits may play a role in wiring events necessary for higher cognitive functions such as social behavior and language. We discuss genetic, electrophysiological, and behavioral evidence that implicates Purkinje cell dysfunction as a major culprit in several diseases and offer a hypothesis as to how canonical cerebellar functions might be at fault in non-motor as well as motor diseases.

## Introduction

The cerebellum is essential for smooth, purposeful movement. Recently, human neuroimaging and animal behavior studies have implicated the cerebellum in the processing of signals for perception, cognition, and emotion (Schmahmann, 2010; Bastian, 2011; D'Angelo and Casali, 2012), particularly in circumstances involving predictions or timing. Participation of the cerebellum in higher order brain function is likely mediated by extensive connections with cortical and sub-cortical centers. These anatomical connections raise the intriguing possibility that cerebellar dysfunction may lead not only to motor impairments, but also to non-motor deficits in complex neurological conditions. Furthermore, the implication that cerebellar circuits malfunction in certain neurodevelopmental disorders suggests that cerebellar processing could be required during development for proper wiring in other brain areas (Kuemerle et al., 2007). We discuss the etiology of cerebellar disease in the context of how circuits are organized, and present evidence that cerebellar connectivity may be altered in ataxia, dystonia, and autism spectrum disorders ASD.

### Although cerebellar circuits are structurally “simple,” they contain millions of connections

**Figure 1:**
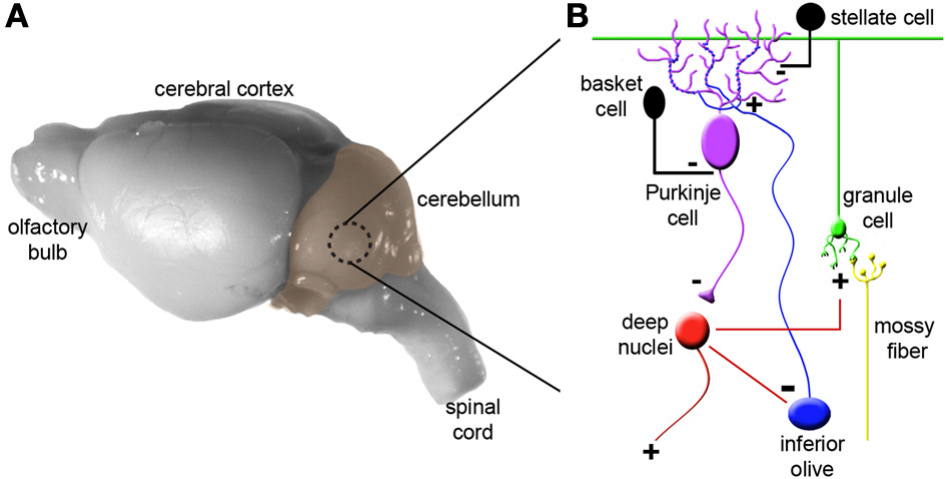
**Cytoarchitecture and connectivity in the cerebellum**. **(A)** Mouse brain shown from a lateral view with the cerebellum highlighted in color. **(B)** The basic cerebellar circuit is comprised of granule cells, Purkinje cells, stellate and basket cell interneurons, and deep nuclei. Afferent information is delivered to the cerebellum as climbing fibers or mossy fibers. The plus and minus signs indicate whether each synapse is excitatory or inhibitory. Note that inhibitory connections between the cerebellar nuclei and inferior olive complete the olivo-cortico-nuclear loop and excitatory projections from the cerebellar nuclei loop back to the cerebellar cortex. Panel **(B)** was modified from (Reeber et al., 2012). For simplicity we have not shown Golgi cells, unipolar brush cells, Lugaro cells, and candelabrum cells.

**Figure 2:**
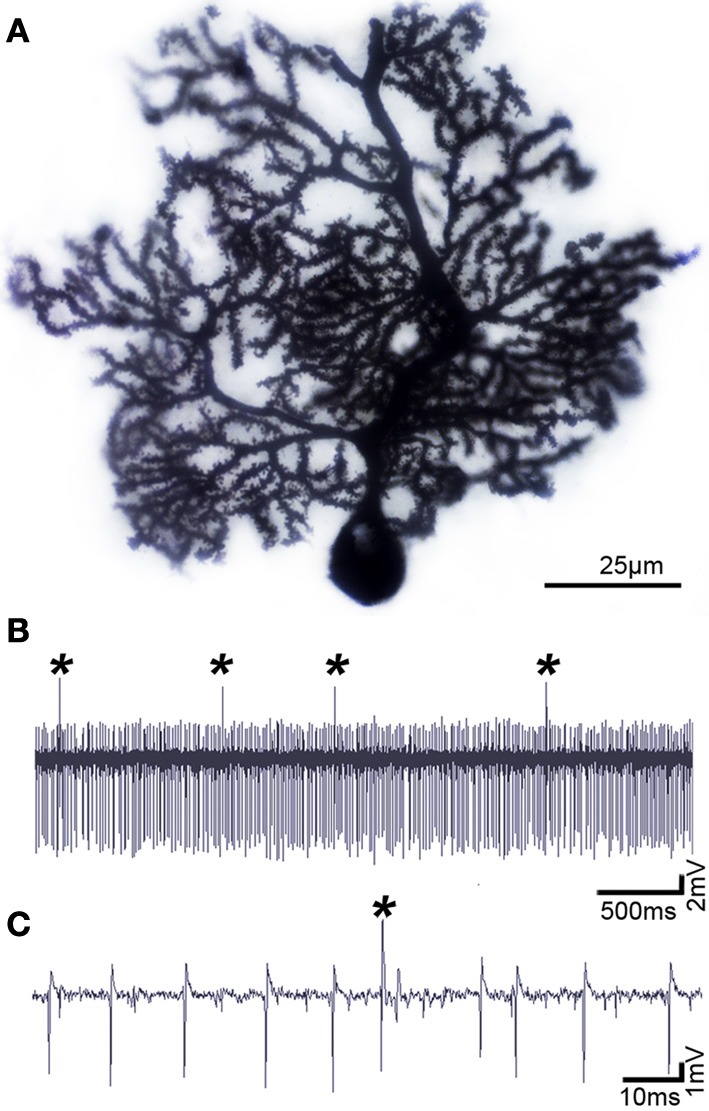
**Purkinje cells have a distinct morphology and electrophysiological profile**. **(A)** Purkinje cell labeled using the classic Golgi-Cox staining method, demonstrating the exquisite morphology and extensive dendritic branching of the Purkinje cell. **(B)** Purkinje cells can be identified by their unique activity: each one fires complex spikes that are triggered by climbing fibers (asterisks) and simples spikes that are driven either by intrinsic activity or by mossy fiber-granule cell inputs. **(C)** Higher power image of the Purkinje cell recording shown in panel **(B)**. Defects in Purkinje cell morphology and/or firing are thought to instigate motor and non-motor neurological conditions.

To appreciate how the cerebellum works, it is useful to first recall the major cell types, and revisit the relationships between them (Figure 1). Purkinje cells are the corner stone of all cerebellar circuits; during development they orchestrate morphogenesis, and in the adult each one computes hundreds of thousands of signals (Figure 1B). The elaborate dendrite of each Purkinje cell is directly innervated by a single excitatory climbing fiber that comes from the inferior olive in the brainstem (Figures 1, 2A). Purkinje cells also receive excitatory input indirectly from mossy fibers, which originate from over two-dozen brain and spinal cord nuclei. Approximately 25 million mossy fibers enter the cerebellum and synapse on ~50 billion granule cells (Palkovits et al., 1972; Andersen et al., 1992). Granule cells then converge massively (100,000 to 1) onto the dendrites of Purkinje cells. This striking expansion from mossy fibers to granule cells and equally striking contraction from granule cells onto the Purkinje cell dendrite is believed to provide a computational benefit, namely the ability of the cerebellum to discriminate a large number of different patterns (Marr, 1969; Albus, 1971; Brunel et al., 2004). Various inhibitory interneurons regulate the excitatory inputs onto Purkinje cells (Figure 1), and specialized astrocytes called Bergmann glia maintain efficient synaptic signaling. The Purkinje cells send exclusively inhibitory signals to the cerebellar nuclei, which control the final output of the cerebellum (White and Sillitoe, 2013). An excitatory feedback projection terminating in mossy fiber-like endings exists between the cerebellar nuclei and granule cell layer, and an inhibitory feedback connection is made from the cerebellar nuclei to the inferior olive. These two connections form parts of the nucleo-cortical (Tolbert et al., 1976; Hess, 1982) and olivo-cortico-nuclear loops (Angaut and Sotelo, 1987; Chaumont et al., 2013), respectively. This canonical cerebellar circuit, which was once thought to be simple and synonymous with motor signaling, is now thought to have underlying complexities that also mediate non-motor brain behaviors.

### Cerebellar connections are integrated into multiple brain networks

**Figure 3:**
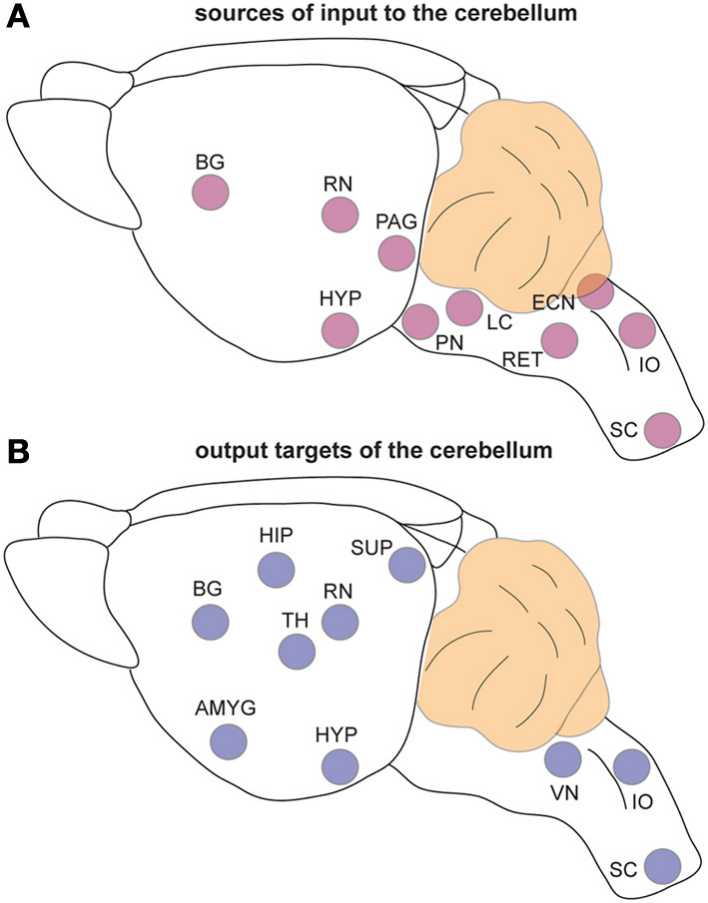
**The cerebellum is extensively connected to the brain and spinal cord. (A)** Schematic representation of brain regions that send input to the cerebellum. **(B)** Schematic representation of the regions that receive information from the cerebellum. Note that the TH is a major relay station for cerebellar input to the cortex while the PN is the primary gateway for cerebral cortical input to the cerebellum. Abbreviations: AMG, amygdala; BG, basal ganglia; ECN, external cuneate nucleus; HIP, hippocampus; HYP; hypothalamus; IO, inferior olive; LC, locus coeruleus; PAG, periaqueductal gray; PN, pontine nuclei; RET, reticular nucleus; RN, red nucleus; SC, spinal cord; SUP, superior colliculi; TH, thalamus; VN, vestibular nuclei.

The classic view that cerebellar function is restricted to controlling motor coordination has been challenged by recent imaging studies in humans that suggest cerebellar contributions to cognition (language), emotional behavior (fear), sleep, and even non-somatic, visceral responses (Demirtas-Tatlidede et al., 2011; Baumann and Mattingley, 2012; D'Angelo and Casali, 2012). Anatomical studies performed in non-human primates and rodents strongly support the imaging data. Extensive mono- and poly-synaptic pathways connect the cerebellum to the cerebral cortex, hippocampus, amygdala, hypothalamus, periaqueductal gray, basal ganglia, thalamus, brain stem, and spinal cord (Dietrichs and Haines, 1989; Middleton and Strick, 2001; Hoshi et al., 2005; Cerminara et al., 2009; Buckner et al., 2011; Dum and Strick, 2012). Considering such widespread connections between the cerebellum and the forebrain, dozens of brainstem nuclei, and with several major autonomic centers (Figure 3), it seems difficult to imagine that cerebellar circuit dysfunction would interfere only with the ability to perform motor tasks. Still, valid arguments against non-motor contributions of the cerebellum have been presented (Glickstein, 2007), and recent data demonstrates that caution should be taken when interpreting for cerebellar non-motor behavior in experimental preparations (Galliano et al., 2013). Keeping in mind that a lively debate continues as to whether the cerebellum is involved in non-motor function (Lemon and Edgley, 2010), in the following sections we present recent evidence that has unveiled unexpected roles for the cerebellum in conditions that are historically “non-cerebellar” (Table 1).

**Table 1:** **Cerebellar dysfunction contributes to motor and non-motor diseases**.

**MOTOR DISEASES**
Ataxia
Dystonia
Huntington's
Multiple sclerosis
Parkinson's
Tourette's (and other “tic”-related disorders)
Tremor
**NON-MOTOR DISEASES**
Autism spectrum disorders
Dyslexia
Fetal alcohol syndrome
Medulloblastoma
Obsessive-compulsive disorder
Schizophrenia
Sleep apnea
Vertigo

The list is by no means exhaustive, although it does include some major conditions with either known (e.g., ataxia) or heavily suspected (e.g., autism spectrum disorders) cerebellar involvement. Note that in each section the different diseases are listed in alphabetical order. And, some motor disorders may have additional complex non-motor symptoms (e.g., mental retardation in ataxia) and vice versa, some patients with non-motor disorders have problems making day-to-day movements (e.g., motor abnormalities in autism spectrum disorders).

#### Diseases of the cerebellum: significantly more than just uncoordinated locomotion

Cerebellar damage causes a number of motor symptoms including dysmetria (in which patients overshoot (hypermetria) or undershoot (hypometria) a target during voluntary goal-directed tasks), hypotonia, tremor, and dysarthic speech. These symptoms, and the interpretations of what they mean to brain function date back to the pioneering neurological examinations of Sir Gordon Holmes (Holmes, 1939). However, descriptions of cerebellar diseases, and in particular those that affect Purkinje cell development and/or function, typically disrupt the accuracy and coordination of movement, conditions which are often cumulatively referred to as “ataxia.”

### Ataxia, the classic case of cerebellar dysfunction

As a symptom, ataxia refers to uncoordinated movement and as a disorder it refers to a family of neurological diseases that typically involve neurodegeneration. Ataxia-related defects can also be acquired, and develop as a result of stroke, multiple sclerosis, tumors, alcoholism, peripheral neuropathy, metabolic disorders, and vitamin deficiencies (Klockgether, 2010). Ataxia can also arise sporadically (Klockgether, 2010). Patients with ataxia have poor muscle control, and when they have limb movement problems the lack of balance and coordination ultimately disturbs their gait, a symptom often associated with cerebellar defects.

Cerebellar ataxia is the most common form of ataxia. There are over 60 forms of inherited cerebellar-based ataxia, with more than half of them classified as either spinocerebellar ataxias, Friedreich's ataxia, episodic ataxia, or fragile X tremor/ataxia syndrome (Durr, 2010; Klockgether, 2010). Recapitulating disease mechanisms in engineered animal models has allowed major breakthroughs in our understanding of the pathogenesis of the cerebellar ataxias (Burright et al., 1995; Hamilton et al., 1996; Serra et al., 2006). A unifying cellular phenotype observed in the nervous system of ataxic mice and humans, regardless of the type of ataxia, is extensive Purkinje cell degeneration. However, while neuronal degeneration may be essential for the severe pathophysiological features of ataxia (Chen et al., 2008; Liu et al., 2009), electrophysiological and calcium imaging data show that Purkinje cells and their major inputs are dysfunctional prior to degeneration in ataxia mouse models coinciding with milder ataxic phenotypes (Barnes et al., 2011; Hourez et al., 2011; Shakkottai et al., 2011; Kasumu et al., 2012; Hansen et al., 2013). This prompts two considerations regarding disease etiology in any disorder that alters the brain at the level of circuits: (1) neuronal function can be affected in the absence of pathological defects, and (2) neuronal circuit dysfunction may be the primary cause of behavioral symptoms. A case in point is dystonia, where no clear or consistent pathology is evident, yet brain dysfunction can cause overt behaviors that are obstructive to daily life.

### Dystonia pathogenesis provides new insights into cerebellar (dys)connectivity

Dystonia is a complex movement disorder that causes involuntary, sustained muscle contractions that result in postural twisting and repetitive movements (Hallett, 2009; Shamim et al., 2011). Symptoms can be mild and transient, appearing only under conditions of exertion or fatigue, or severe and constant enough to make even simple day-to-day movements impossible. The involuntary painful muscle contractions can affect virtually any muscle in the body, causing blepharospams in the eyelids [a frequent result of anti-psychotic drugs; (Hallett, 2009)], to the common writer's cramp (Shamim et al., 2011), to inherited torsion dystonia that blocks the normal execution of trunk and limbs movements (Muller, 2009; Shamim et al., 2011). It can be acquired as a hereditary disorder, or spontaneously arise as an idiopathic condition. Although it is considered the third most common motor disease, the true prevalence of dystonia is difficult to estimate because it can be comorbid with other disorders such as Parkinson's disease, Huntington's disease, stroke, or ataxia, and many milder cases do not get reported (Muller, 2009; Asmus and Gasser, 2010). Despite the wide range of its manifestations and causes, dystonia consistently involves erroneous communication along circuits that link the cerebral cortex, basal ganglia, thalamus, and brainstem (Hendrix and Vitek, 2012). Recently, several groups have considerably deepened our understanding of dystonia by confirming that the cerebellum can play a role in the disorder (Argyelan et al., 2009; Calderon et al., 2011; LeDoux, 2011; Table 2). Now, the general consensus in field is that in dystonia, communication is disrupted along two primary pathways: the cerebello-thalamo-striatial (CTS) circuit and cerebello-thalamo-cortical (CTC) circuit (Niethammer et al., 2011). Moreover, a recent elegant study demonstrated using a model of rapid onset dystonia-parkinsonism that defects in either the basal ganglia or the cerebellum could instigate disease onset (Calderon et al., 2011; Table 2).

**Table 2:** **Animal models and human data that implicate the cerebellum in dystonia**.

**A. ANIMAL MODELS**			
**Model**	**Mode of induction**	**Contribution**	**Reference**
Genetically *dystonic rat (dt)*	Spontaneous mutation in the *Atcay* gene	Purkinje cell and cerebellar nuclei “burst” firing	LeDoux et al., 1993, 1998; LeDoux and Lorden, 2002
*tottering* mice	Spontaneous mutation in the gene encoding the alpha subunit of the Cacna1a P/Q-type calcium channel	Purkinje cells might contribute to dystonia	Campbell et al., 1999; Neychev et al., 2008
Purkinje specific deletion of Cacna1a	Conditional mouse genetics	Regional Purkinje cell dysfunction initiates dystonia	Raike et al., 2012
*Dyt1* mutant mice	Genetically engineered knock-in into *Tor1a*	Gene dysfunction in cerebellum may cause dystonia	Ulug et al., 2011; Yokoi et al., 2012
Kainic acid (glutamate receptor agonist)	Injection into cerebellum	Abnormal cerebellar activity can induce dystonia	Pizoli et al., 2002
Ouabain (binds and inhibits the Na^+^/K^+^-ATPase sodium pump)	Micro-pump infusion into cerebellum	Cerebellum (presumably Purkinje cells) instigates dystonia	Calderon et al., 2011
**B. HUMAN PHYSIOLOGY AND NEUROPATHOLOGY**			
**Approach**	**Measurement**	**Contribution**	**Reference**
Eye blink conditioning (cervical dystonia)	Function of the olivo-cerebellar pathway	Functional defects in the cerebellar circuit in dystonia	Teo et al., 2009
DTI imaging (DYT1 and DYT6 carriers)	Tractography	Cerebello-thalamic pathway is defective in dystonia patients	Argyelan et al., 2009
[(18)F]-fluorodeoxyglucose PET (DYT11 myoclonus-dystonia patients)	Metabolic changes	Metabolic changes in the cerebellum and inferior olive of dystonia patients	Carbon et al., 2013
Neuropathology (cervical dystonia)	Purkinje cell density	Purkinje cell loss is “patchy” in dystonia	Prudente et al., 2013

This table lists some recent publications that demonstrate a potentially critically role for the cerebellum is various forms of dystonia. For clarity we have only listed a few pertinent examples.

Functional imaging studies have revealed abnormal cerebellar activity in *DYT1* dystonia (Eidelberg et al., 1998), hemi-dystonia (Ceballos-Baumann et al., 1995), exercise-induced paroxysmal dystonia (Kluge et al., 1998), writer's cramp (Odergren et al., 1998; Preibisch et al., 2001), cervical dystonia (Galardi et al., 1996) and blepharospasm (Hutchinson et al., 2000). Consistent with these human data, abnormal cerebellar activity is observed in several genetic models of dystonia, including dystonic (*dt*) rats, both transgenic and knock-in *Dyt1* mice, and spontaneous mutant mice such as *tottering* (Brown and Lorden, 1989; Campbell and Hess, 1998; Calderon et al., 2011; Ulug et al., 2011; Zhao et al., 2011). *In vivo* electrophysiology recording in these rodent models reveals that Purkinje cells lose their regular firing patterns (Figures 2B,C) and instead fire in erratic “burst” patterns (LeDoux, 2011). Interestingly, in mice with ataxia it has been suggested that irregular firing of Purkinje cells is the primary alteration that causes motor defects (Walter et al., 2006). Surgical removal of the cerebellum terminates the dystonic attacks in rodents, supporting the notion that the cerebellum can drive dystonia (Neychev et al., 2008; LeDoux, 2011; Neychev et al., 2011). At the cellular level, Ellen Hess and colleagues have pioneered the view that cerebellar Purkinje cells may be the source of dystonia (Campbell et al., 1999). In their initial experiments they removed Purkinje cells by cross-breeding dystonic *tottering* mice with mutants that exhibit Purkinje cell degeneration (Campbell et al., 1999). Remarkably, doing so alleviated dystonia. In their recent studies, Hess' group showed using an elegant conditional genetic approach that selectively eliminating the Cacna1a calcium channel in Purkinje cells is sufficient to evoke widespread dystonic movements (Raike et al., 2012). Moreover, this conditional approach was further used to show that stress, caffeine, and alcohol can operate through shared mechanisms to trigger severe episodic dystonia attacks (Raike et al., 2013). While Purkinje cell defects induce dystonia, extra-cerebellar synapses may be targeted to block dystonia. Micro-lesions made in the central-lateral thalamus, which connects the cerebellum to the basal ganglia (Ichinohe et al., 2000), alleviated motor defects in mice with rapid-onset dystonia-parkinsonism (Calderon et al., 2011).

On the one hand, genetically altering Purkinje cells has revealed an unexpected requirement for the cerebellum in dystonia, yet on the other hand, it may not be entirely surprising that altering Purkinje cell function would produce complex motor deficits. In fact, one has to wonder whether defective Purkinje cell communication could also, and perhaps simultaneously, influence non-motor behavior. This logic was recently put to the test in experiments that sought to determine whether the cerebellum is linked to ASD.

### Autism spectrum disorders may be linked to cerebellar development and function

The ASD's are developmental disorders characterized by impaired social communication, repetitive stereotypic behaviors, and delayed language development (Association, 1994). Individuals with ASD can also display dysfunction in both fine and gross motor skills (Fatemi et al., 2012). Although the signs and symptoms of ASD have become well appreciated, an ongoing debate has been centered on one important question: what regions of the brain are defective in ASD? Not so appreciated is that the cerebellum exhibits consistent neuropathological abnormalities in ASD (Ritvo et al., 1986; Bauman, 1991). In postmortem brain tissue from ASD patients, regardless of age, sex, and cognitive ability, a significant decrease in the number of Purkinje cells is reported (Bauman and Kemper, 2005; Whitney et al., 2008; Fatemi et al., 2012). In addition, functional neuroimaging demonstrates abnormal cerebellar activation in patients with ASD (Allen et al., 2004). Although controversial, in part due to co-morbidity with other developmental deficits, magnetic resonance imaging has also revealed hypoplasia of the cerebellum in some ASD patients (Courchesne et al., 1994; Stanfield et al., 2008; Scott et al., 2009).

Genetic studies also support a role for the cerebellum in ASD (Fatemi et al., 2012). For example, trinucleotide repeat expansions that cause fragile X syndrome by disrupting the function of the gene *FMR1* lead to cerebellar vermis abnormalities. In both global and Purkinje cell-specific fragile X knockout mice, Purkinje cell spine morphology, synaptic plasticity, and cerebellar behaviors are impaired. Moreover, in humans, cerebellar learning is deficient as fragile X patients show abnormal eye blink conditioning (Koekkoek et al., 2005; Smit et al., 2008; Tobia and Woodruff-Pak, 2009). There is also a Fragile X associated ataxia/tremor syndrome exhibited by parents of Fragile X patients (Hagerman et al., 2001; Hall and O'Keefe, 2012). This syndrome is linked to “premutation” expansions in the fragile X gene and presents with classic cerebellar deficits of gait ataxia and tremor. Imaging studies show clear atrophy of the cerebellum.

Other genes highly expressed in cerebellum such as *EN2, MET*, and *GABRB3* may also be associated with non-syndromic ASD. Each of these genes exhibits specific roles during cerebellar development. In mice, *En2* is required for cell proliferation, tissue patterning, regional morphogenesis, and circuit formation in the cerebellum (White and Sillitoe, 2013). Importantly, two intronic polymorphisms in human *EN2* have been reported to be associated with the risk of developing ASD (Gharani et al., 2004; Wang et al., 2008; Banerjee-Basu and Packer, 2010; Sen et al., 2010; Yang et al., 2010). Loss of *En2* in mice results in altered aggressiveness and excessive grooming, which are hallmark ASD-like behaviors (Cheh et al., 2006; Brielmaier et al., 2012). Several studies have demonstrated an association between three *MET* single nucleotide polymorphisms and ASD (Campbell et al., 2008; Hedrick et al., 2012). MET is expressed in proliferating granule cell precursors and disrupting its function results in cerebellar hypoplasia (Ieraci et al., 2002; Fatemi et al., 2012). Positive associations with ASD have also been reported for both common and rare variants of the *GABRB3* gene (Banerjee-Basu and Packer, 2010), and *GABRB3* expression is reduced in the cerebellum of affected individuals. Importantly, *GABRB3* is located within chromosome 15q11–13, a site linked to duplications that are associated with ASD (Fatemi et al., 2012). *GABRB3* null mice display hypoplasia of the cerebellar vermis (DeLorey et al., 2008; Fatemi et al., 2009, 2012). Despite the potential links between cerebellar dysfunction and ASD pathogenesis, no clear view has emerged about *why* the cerebellum might be involved in ASD (genetic and cellular mechanisms?) or *how* it might be involved (specific brain connections or neural circuits?). However, recent landmark studies strongly support the idea that Purkinje cell dysfunction can result in ASD.

Tuberous sclerosis (TSC1, TSC2) is a rare disorder associated with ASD, and is characterized by benign tumors (harmartomas) that form in the brain, skin, eyes, kidneys, and heart (Curatolo et al., 2008). Interestingly, tuberous sclerosis patients with cerebellar lesions have more severe ASD symptoms than patients with lesions in only other brain regions (Eluvathingal et al., 2006). In a recent paper, Sahin and co-workers showed that loss of *Tsc1* from cerebellar Purkinje cells is sufficient to cause social impairments, cognitive defects, abnormal vocalizations, and a number of motor problems (Tsai et al., 2012). The mutant mice also exhibited a reduction in the number of Purkinje cells, and an increase in the expression of endoplasmic reticulum and oxidative stress response markers. The study further showed that Purkinje cell excitability was altered in both heterozygous and homozygous *Tsc1* mutants (Tsai et al., 2012) in a very similar manner as has been described in spinocerebellar ataxia models (Hourez et al., 2011; Shakkottai et al., 2011; Kasumu et al., 2012; Hansen et al., 2013). Both the pathology and abnormal ASD-like behaviors were successfully blocked in mutants treated with the mTOR inhibitor rapamycin. Together, these *Tsc1* conditional mutants recapitulated several core features of human ASD and using their model the authors demonstrate that pharmacological treatments that target Purkinje cell function can alleviate multiple ASD-associated features (Tsai et al., 2012). In a different study, loss of *Tsc2* in Purkinje cells resulted in neurodegeneration, increased repetitive behavior, and social interaction deficits (Reith et al., 2013). Cumulatively, the impressive body of data from these two studies suggests that Purkinje cell dysfunction can cause ASD-like behaviors, and that defects in specific cerebellar circuits might be sufficient to trigger downstream neuronal network alterations that contribute to severe abnormalities in motor and non-motor behaviors.

#### Might the cerebellum be performing a common computational task in its motor and non-motor functions?

**Figure 4:**
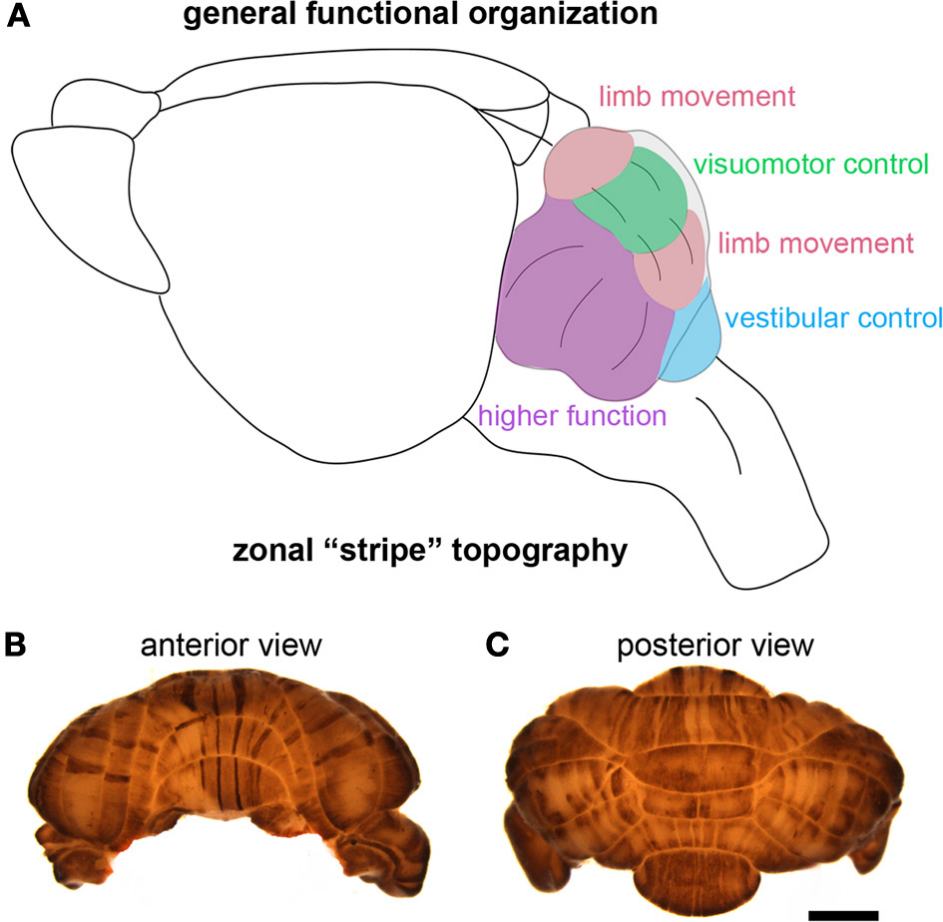
**The cerebellum is highly compartmentalized into functional regions. (A)** Schematic illustrating the division of the cerebellum into behaviorally relevant domains. The cartoon is a simplified model of the functional cerebellum and is based on functional imaging, human and animal lesions, afferent connectivity, electrophysiology, and animal behavior studies. **(B,C)** Wholemount immunohistochemical staining of the mouse cerebellum—zebrin II expression reveals the intricate patterning of the cerebellum into zones. The scale bar in **(C)** = 2 mm (also applies to **(B)**.

Although the identification of the Purkinje cell as a major player in motor and non-motor disease has opened new avenues for understanding complex brain disorders (Table 3), one question that immediately arises is how could a single cell type with seemingly unique and specialized functions encode such diverse disease-related information? One can speculate that some of the basic computational capacities of the cerebellum that have been studied with respect to motor behavior, such as the ability to discriminate patterns and the capacity to use these patterns to learn to make context-dependent predictions (Bastian, 2011), are useful to non-motor areas of the brain. For example, it is intriguing to consider whether cerebellar output may be required during a critical period of development so that cortical circuits responsible for complex social behavior and language can be properly wired. This may not be confined to cortical circuitry. Interestingly, Herrup and colleagues observed that loss of *En2* resulted in defects in the position of the amygdala, a brain region regularly altered in individuals with ASD (Kuemerle et al., 2007). These findings suggest that a gene (*En2*) expressed exclusively in the mid/hindbrain region can affect distant cerebral cortex structures such as the amygdala. Herrup and colleagues hypothesized that disrupting the location of neurons relative to their efferent and afferent partners may have detrimental effects on cognition. In this scenario, cerebellar dysfunction during development could result in ASD-like symptoms (Kuemerle et al., 2007). In adults who suffer cerebellar damage, cognitive impairments may result from a loss of cerebellar processing power contributing to tasks involving prediction or complex sensory discrimination (Bastian, 2011). Such hypotheses derive support from the parallel anatomical substrates that connect the cerebellum to motor areas and non-motor areas. Progress on these fascinating questions is likely to emerge from studies of the remarkable patterning of the cerebellum into a complex array of topographic “zonal” circuits that are thought to shape cellular function during behavior (Figure 4).

**Table 3:** **Animal models of cerebellar dysfunction**.

**Model**	**Phenotype**	**Relationship to disease**
ATXN1[82Q]	ataxia	Spinocerebellar ataxia type 1
ATXN2[Q127]	ataxia	Spinocerebellar ataxia type 2
Genetically *dystonic rat (dt)*	Cerebellar functional defects and severe co-contractions of the muscles	Dystonia
*tottering* mice	Baseline locomotor dysfunction with stress induced increase	Episodic ataxia and dystonia
Purkinje specific deletion of Cacna1a	Ataxia and dystonic-like postures	Dystonia and ataxia
*Dyt1* mutant mice	Generalized motor dysfunction	Hereditary dystonia
Kainic acid (glutamate receptor agonist)	Dystonic postures of the limbs and trunk	Generalized dystonia
Ouabain (binds and inhibits the Na^+^/K^+^-ATPase sodium pump)	Ataxia and dystonic-like postures (hyperextended limbs)	Rapid onset dystonia-Parkinsonism
*En2* null mice	Motor coordination, motor learning, and social behavior deficits	Autism spectrum disorders
*Met* knock-in mutant mice	Cerebellar development defects	Autism spectrum disorders
*Gabrb3* null mice	Cerebellar morphogenesis defects and social behavior impairments	Autism spectrum disorders
Purkinje cell deletion of *Tsc1*	Purkinje cell electrophysiological dysfunction, repetitive behaviors, social behavior abnormalities	Autism spectrum disorders
Purkinje cell deletion of *Tsc2*	Social behavior defects and repetitive behaviors	Autism spectrum disorders

This table lists the animal models we have discussed and their utility in understanding specific cerebellar diseases. Note that for clarity only two spinocerebellar ataxia (SCA) models are mentioned—SCA has been extensively studied using different models.

#### Toward a circuit topography hypothesis for understanding cerebellar disease

Natural selection has adorned the animal kingdom with a rich collection of exquisite patterns. We revel in admiration of butterfly wing spots, peacock feathers and zebra stripes. Beyond their beauty, these precise patterns are essential for sexual selection and evading predators. In humans, our own body parts such as ribs, vertebrae, and digits develop into patterns that permit the execution of essential day-to-day functions. The human brain, arguably the most complicated structure in nature, is no exception to the hypothesis that patterns are inherent to all forms, regardless of their simplicity or complexity. Much like the developing wings and body segments of an insect, the mammalian brain contains a striking array of patterns. Each of the billions of neurons in the human brain is decorated with a specific pattern of connections that drive brain function. Although many regions of the brain have patterned neural circuits (Reeber et al., 2012), the cerebellum arguably contains the most exquisitely patterned circuits of all central nervous system structures. This high level of patterning may be essential for packaging a large number of functionally distinct circuits into a logical network for seamless communication during behavior.

Cerebellar circuits are patterned into a topographic map of genetically determined “zones” (White and Sillitoe, 2013). Zones are best revealed by molecular expression patterns in Purkinje cells. The most comprehensively studied zonal marker is zebrin II (Brochu et al., 1990) (Figures 4B,C), also known as aldolase C. Zebrin II is expressed by a subset of Purkinje cells (zebrin II +) that alternate with Purkinje cells that do not express zebrin II (zebrin II −), thus forming complementary rows of biochemically distinct Purkinje cells. The zonal organization of zebrin II is symmetrical about the midline, highly reproducible between individuals, and conserved across species (Sillitoe et al., 2005). Molecular tools such as zebrin II expression have been used to show that zonal compartments divide the cerebellar cortex into thousands of reproducible units, with each one containing several hundred Purkinje cells (Apps and Hawkes, 2009). The Purkinje cell zonal plan has a predictable and well-defined relationship to its thousands of incoming afferent projections [Figure 3; (Reeber et al., 2012)]. Moreover, each cerebellar cortical region has a defined and strict relationship to specific cells within the cerebellar nuclei, which send topographic projections out of the cerebellum to unique regions within the brain and spinal cord [(Uusisaari and De Schutter, 2011); Figure 3]. Together, Purkinje cell zones and their associated synapses organize cerebellar function into a spatial map that encodes multiple behaviors (Wadiche and Jahr, 2005; Horn et al., 2010; Cerminara and Apps, 2011). It is intriguing to speculate that this zonal plan may extend beyond cerebellar circuits into connected regions such as the thalamus and cerebral cortex. Indeed, cerebellar efferent projections to the inferior olive and the basal ganglia are highly topographic, and because of bi-directional connectivity projections between these structures forms closed loop circuits (Middleton and Strick, 2000; Bazzigaluppi et al., 2012).

With such a high level of organization it seems plausible that certain circuits, and therefore certain zones, may be more affected by some diseases and not others. That is, could disrupting one set of zones cause ataxia while disrupting an adjacent set cause dystonia? Perhaps. However, given that each zone likely encodes multiple behaviors (Cerminara and Apps, 2011), manipulating the function of any given set would almost certainly result in a “mixed” disease outcome. The phenotypes of several animal models of episodic movement disorders support this idea. For instance, in *Cacna1a* mutant mice [*tottering*; (Alvina and Khodakhah, 2010)], loss of the Cav2.1 voltage dependent calcium channel not only causes ataxic episodes, but severe dystonia can also be induced in the same mice. Similarly, in *Tsc1, Tsc2*, and *En2* mouse models of ASD, loss of cerebellar function triggers circuit defects that disrupt both motor and non-motor behaviors. And, *En2* mutant mice exhibit severe alterations in Purkinje cell patterning and consequently, at least three functionally distinct classes of afferent fibers are mis-targeted into ectopic positions within the cerebellar cortex (White and Sillitoe, 2013). It is therefore intriguing that Purkinje cell loss may be patterned in dystonia and ASD since the cell loss has been described as “patchy” in both conditions (Carper et al., 2006; Prudente et al., 2013). The burning question that we must now tackle is does zonal function directly control disease related behaviors? The answer may lie within the operational units of the zones, which are referred to as cerebellar modules (Ruigrok, 2011). Each module is comprised of topographically organized afferent fibers, Purkinje cell stripe gene expression (e.g., zebrin II), and the zonally organized Purkinje cell efferent projections to the cerebellar nuclei. Systematic analyses will have to be conducted in existing mutant mouse models in order to determine how each module, or subsets of functionally related modules, operates during the expression of disease-related behaviors. In addition, however, powerful inducible approaches such as channelrhodopsin or CreER genetics should be used to target specific modules (or specific circuits within them) to ask whether defective connectivity in select pathways can recapitulate the disease phenotypes. Such models would be invaluable for therapeutic design and testing. In parallel, further studies in humans should be conducted. Recent likelihood meta-analysis of neuroimaging data demonstrated a precise functional topography in different lobules; subsets of lobules are apparently associated with specific functions (e.g., lobule V = sensorimotor function and lobule VII = cognitive function; (Stoodley and Schmahmann, 2009). Each set of lobules was also linked to specific cerebello-cerebral cortical loops (Stoodley and Schmahmann, 2010), which include connections with somatomotor, premotor and association cortices (Buckner et al., 2011). In general, these functional and anatomic topographies are remarkably reminiscent of the transverse divisions that were delineated by mouse developmental and genetic analyses of zones (Ozol et al., 1999; Sgaier et al., 2007). For more than 60 years zonal cerebellar circuits have been invaluable for understanding basic neuroanatomy, development, cellular function, and behavior. Now, the time is right to apply this wealth of knowledge toward understanding the cellular and molecular mechanisms of neurological disease using animal models and also human pathophysiology. Purkinje cell zones are highly attractive candidates for mediating cerebellar disease (Tolbert et al., 1995; Sarna and Hawkes, 2003). In this context, the expression of calcium associated proteins such as mGluR, EAAT4, and IP3R1 may render certain zones more vulnerable to particular insults (Welsh et al., 2002; Wadiche and Jahr, 2005; Schorge et al., 2010). Although, why particular zones are so susceptible to specific conditions whereas others are resistant, remains an intriguing mystery.

#### Summary

Despite the remarkable strides that have been made in understanding the role of the cerebellum in normal brain behavior and disease, we still do not have a consensus on either issue. We are far from fully understanding what the cerebellum does or what happens if it fails to work properly. Moreover, with only five major neuronal types, one wonders how diverse information from regions ranging from the spinal cord and brainstem to the hypothalamus and basal ganglia converge within functionally coherent circuits in the cerebellum. Even after more than 100 years since the seminal work of Cajal, the cerebellum remains one of the most intriguing structures in the body: despite its simple structure it has the computing power to process more information than any other brain region, and changes to its normal state translate into devastating conditions that apparently reverberate throughout the brains major circuits. While the precise mechanisms that mediate cerebellar (dys)function remain a mystery, powerful new genetic and circuit physiology approaches hold great promise for improving our understanding of this fascinating brain region.

### Conflict of interest statement

The authors declare that the research was conducted in the absence of any commercial or financial relationships that could be construed as a potential conflict of interest.

## References

[B1] Albus. (1971). A theory of cerebellar function. Math. Biosci. 10, 25–61 10.1016/0025-5564(71)90051-4

[B2] Allen G. Muller R. A. Courchesne E. (2004). Cerebellar function in autism: functional magnetic resonance image activation during a simple motor task. Biol. Psychiatry 56, 269–278 10.1016/j.biopsych.2004.06.005 15312815

[B3] Alvina K. Khodakhah K. (2010). KCa channels as therapeutic targets in episodic ataxia type-2. J. Neurosci. 30, 7249–7257 10.1523/JNEUROSCI.6341-09.2010 20505091 PMC2909841

[B4] Andersen B. B. Korbo L. Pakkenberg B. (1992). A quantitative study of the human cerebellum with unbiased stereological techniques. J. Comp. Neurol. 326, 549–560 10.1002/cne.903260405 1484123

[B5] Angaut P. Sotelo C. (1987). The dentato-olivary projection in the rat as a presumptive GABAergic link in the olivo-cerebello-olivary loop. An ultrastructural study. Neurosci. Lett. 83, 227–231 10.1016/0304-3940(87)90090-5 3441304

[B6] Apps R. Hawkes R. (2009). Cerebellar cortical organization: a one-map hypothesis. Nat. Rev. Neurosci. 10, 670–681 10.1038/nrn2698 19693030

[B7] Argyelan M. Carbon M. Niethammer M. Ulug A. M. Voss H. U. Bressman S. B. (2009). Cerebellothalamocortical connectivity regulates penetrance in dystonia. J. Neurosci. 29, 9740–9747 10.1523/JNEUROSCI.2300-09.2009 19657027 PMC2745646

[B8] Asmus F. Gasser T. (2010). Dystonia-plus syndromes. Eur. J. Neurol. 17 Suppl. 1, 37–45 10.1111/j.1468-1331.2010.03049.x 20590807

[B9] Association A. P. (1994). Diagnostic and Statistical Manual of Mental Disorders (DSM-4), 4th Edn. Washington, DC: APA

[B10] Banerjee-Basu S. Packer A. (2010). SFARI Gene: an evolving database for the autism research community. Dis. Model. Mech. 3, 133–135 10.1242/dmm.005439 20212079

[B11] Barnes J. A. Ebner B. A. Duvick L. A. Gao W. Chen G. Orr H. T. (2011). Abnormalities in the climbing fiber-Purkinje cell circuitry contribute to neuronal dysfunction in ATXN1[82Q] mice. J. Neurosci. 31, 12778–12789 10.1523/JNEUROSCI.2579-11.2011 21900557 PMC3178465

[B12] Bastian A. J. (2011). Moving, sensing and learning with cerebellar damage. Curr. Opin. Neurobiol. 21, 596–601 10.1016/j.conb.2011.06.007 21733673 PMC3177958

[B13] Bauman M. L. (1991). Microscopic neuroanatomic abnormalities in autism. Pediatrics 87, 791–796 2020538

[B14] Bauman M. L. Kemper T. L. (2005). Neuroanatomic observations of the brain in autism: a review and future directions. Int. J. Dev. Neurosci. 23, 183–187 10.1016/j.ijdevneu.2004.09.006 15749244

[B15] Baumann O. Mattingley J. B. (2012). Functional topography of primary emotion processing in the human cerebellum. Neuroimage 61, 805–811 10.1016/j.neuroimage.2012.03.044 22465459

[B16] Bazzigaluppi P. Ruigrok T. Saisan P. De Zeeuw C. I. de Jeu M. (2012). Properties of the nucleo-olivary pathway: an *in vivo* whole-cell patch clamp study. PloS ONE 7:e46360 10.1371/journal.pone.0046360 23029495 PMC3459892

[B17] Brielmaier J. Matteson P. G. Silverman J. L. Senerth J. M. Kelly S. Genestine M. (2012). Autism-relevant social abnormalities and cognitive deficits in engrailed-2 knockout mice. PloS ONE 7:e40914 10.1371/journal.pone.0040914 22829897 PMC3400671

[B18] Brochu G. Maler L. Hawkes R. (1990). Zebrin II: a polypeptide antigen expressed selectively by Purkinje cells reveals compartments in rat and fish cerebellum. J. Comp. Neurol. 291, 538–552 10.1002/cne.902910405 2329190

[B19] Brown L. L. Lorden J. F. (1989). Regional cerebral glucose utilization reveals widespread abnormalities in the motor system of the rat mutant dystonic. J. Neurosci. 9, 4033–4041 2585066 10.1523/JNEUROSCI.09-11-04033.1989 PMC6569939

[B20] Brunel N. Hakim V. Isope P. Nadal J. P. Barbour B. (2004). Optimal information storage and the distribution of synaptic weights: perceptron versus Purkinje cell. Neuron 43, 745–757 15339654 10.1016/j.neuron.2004.08.023

[B21] Buckner R. L. Krienen F. M. Castellanos A. Diaz J. C. Yeo B. T. (2011). The organization of the human cerebellum estimated by intrinsic functional connectivity. J. Neurophysiol. 106, 2322–2345 10.1152/jn.00339.2011 21795627 PMC3214121

[B22] Burright E. N. Clark H. B. Servadio A. Matilla T. Feddersen R. M. Yunis W. S. (1995). SCA1 transgenic mice: a model for neurodegeneration caused by an expanded CAG trinucleotide repeat. Cell 82, 937–948 10.1016/0092-8674(95)90273-2 7553854

[B23] Calderon D. P. Fremont R. Kraenzlin F. Khodakhah K. (2011). The neural substrates of rapid-onset Dystonia-Parkinsonism. Nat. Neurosci. 14, 357–365 10.1038/nn.2753 21297628 PMC3430603

[B24] Campbell D. B. Hess E. J. (1998). Cerebellar circuitry is activated during convulsive episodes in the tottering (tg/tg) mutant mouse. Neuroscience 85, 773–783 10.1016/S0306-4522(97)00672-6 9639271

[B25] Campbell D. B. Li C. Sutcliffe J. S. Persico A. M. Levitt P. (2008). Genetic evidence implicating multiple genes in the MET receptor tyrosine kinase pathway in autism spectrum disorder. Autism Res. 1, 159–168 10.1002/aur.27 19360663 PMC2678909

[B26] Campbell D. B. North J. B. Hess E. J. (1999). Tottering mouse motor dysfunction is abolished on the Purkinje cell degeneration (pcd) mutant background. Exp. Neurol. 160, 268–278 10.1006/exnr.1999.7171 10630211

[B28a] Carbon M. Raymond D. Ozelius L. Saunders-Pullman R. Frucht S. Dhawan V. (2013). Metabolic changes in DYT11 myoclonus-dystonia. Neurology 80, 385–391 10.1212/WNL.0b013e31827f0798 23284065 PMC3589244

[B28] Carper R. A. Wideman G. M. Courchesne E. (2006). Understanding autism from basic neuroscience to treatment, in Structural Neuroimaging, eds Steve O. Moldin John L. R. Rubenstein (Boca Raton, FL: CRC Press Taylor and Francis), 347–377

[B29] Ceballos-Baumann A. O. Passingham R. E. Marsden C. D. Brooks D. J. (1995). Motor reorganization in acquired hemidystonia. Ann. Neurol. 37, 746–757 10.1002/ana.410370608 7778848

[B30] Cerminara N. L. Apps R. (2011). Behavioural significance of cerebellar modules. Cerebellum 10, 484–494 10.1007/s12311-010-0209-2 20838949 PMC3169775

[B31] Cerminara N. L. Koutsikou S. Lumb B. M. Apps R. (2009). The periaqueductal grey modulates sensory input to the cerebellum: a role in coping behaviour. Eur. J. Neurosci. 29, 2197–2206 10.1111/j.1460-9568.2009.06760.x 19453624

[B32] Chaumont J. Guyon N. Valera A. M. Dugue G. P. Popa D. Marcaggi P. (2013). Clusters of cerebellar Purkinje cells control their afferent climbing fiber discharge. Proc. Natl. Acad. Sci. U.S.A. 110, 16223–16228 10.1073/pnas.1302310110 24046366 PMC3791757

[B33] Cheh M. A. Millonig J. H. Roselli L. M. Ming X. Jacobsen E. Kamdar S. (2006). En2 knockout mice display neurobehavioral and neurochemical alterations relevant to autism spectrum disorder. Brain Res. 1116, 166–176 10.1016/j.brainres.2006.07.086 16935268

[B34] Chen X. Tang T. S. Tu H. Nelson O. Pook M. Hammer R. (2008). Deranged calcium signaling and neurodegeneration in spinocerebellar ataxia type 3. J. Neurosci. 28, 12713–12724 10.1523/JNEUROSCI.3909-08.2008 19036964 PMC2663415

[B35] Courchesne E. Saitoh O. Yeung-Courchesne R. Press G. A. Lincoln A. J. Haas R. H. (1994). Abnormality of cerebellar vermian lobules VI and VII in patients with infantile autism: identification of hypoplastic and hyperplastic subgroups with MR imaging. AJR. Am. J. Roentgenol. 162, 123–130 10.2214/ajr.162.1.8273650 8273650

[B36] Curatolo P. Bombardieri R. Jozwiak S. (2008). Tuberous sclerosis. Lancet 372, 657–668 10.1016/S0140-6736(08)61279-9 18722871

[B37] D'Angelo E. Casali S. (2012). Seeking a unified framework for cerebellar function and dysfunction: from circuit operations to cognition. Front. Neural Circuits 6:116 10.3389/fncir.2012.00116 23335884 PMC3541516

[B38] DeLorey T. M. Sahbaie P. Hashemi E. Homanics G. E. Clark J. D. (2008). Gabrb3 gene deficient mice exhibit impaired social and exploratory behaviors, deficits in non-selective attention and hypoplasia of cerebellar vermal lobules: a potential model of autism spectrum disorder. Behav. Brain Res. 187, 207–220 10.1016/j.bbr.2007.09.009 17983671 PMC2684890

[B39] Demirtas-Tatlidede A. Freitas C. Pascual-Leone A. Schmahmann J. D. (2011). Modulatory effects of theta burst stimulation on cerebellar nonsomatic functions. Cerebellum 10, 495–503 10.1007/s12311-010-0230-5 21132574 PMC3260524

[B40] Dietrichs E. Haines D. E. (1989). Interconnections between hypothalamus and cerebellum. Anat. Embryol. 179, 207–220 10.1007/BF00326585 2644872

[B41] Dum R. P. Strick P. L. (2012). Transneuronal tracing with neurotropic viruses reveals network macroarchitecture. Curr. Opin. Neurobiol. 23, 245–249 10.1016/j.conb.2012.12.002 23287632 PMC3920982

[B42] Durr A. (2010). Autosomal dominant cerebellar ataxias: polyglutamine expansions and beyond. Lancet Neurol. 9, 885–894 10.1016/S1474-4422(10)70183-6 20723845

[B43] Eidelberg D. Moeller J. R. Antonini A. Kazumata K. Nakamura T. Dhawan V. (1998). Functional brain networks in DYT1 dystonia. Ann. Neurol. 44, 303–312 10.1002/ana.410440304 9749595

[B44] Eluvathingal T. J. Behen M. E. Chugani H. T. Janisse J. Bernardi B. Chakraborty P. (2006). Cerebellar lesions in tuberous sclerosis complex: neurobehavioral and neuroimaging correlates. J. Child Neurol. 21, 846–851 10.1177/08830738060210100301 17005099

[B45] Fatemi S. H. Aldinger K. A. Ashwood P. Bauman M. L. Blaha C. D. Blatt G. J. (2012). Consensus paper: pathological role of the cerebellum in autism. Cerebellum 11, 777–807 10.1007/s12311-012-0355-9 22370873 PMC3677555

[B46] Fatemi S. H. Reutiman T. J. Folsom T. D. Thuras P. D. (2009). GABA(A) receptor downregulation in brains of subjects with autism. J. Autism Dev. Disord. 39, 223–230 10.1007/s10803-008-0646-7 18821008 PMC2697059

[B47] Galardi G. Perani D. Grassi F. Bressi S. Amadio S. Antoni M. (1996). Basal ganglia and thalamo-cortical hypermetabolism in patients with spasmodic torticollis. Acta Neurol. Scand. 94, 172–176 10.1111/j.1600-0404.1996.tb07049.x 8899050

[B48] Galliano E. Potters J. W. Elgersma Y. Wisden W. Kushner S. A. De Zeeuw C. I. (2013). Synaptic transmission and plasticity at inputs to murine cerebellar Purkinje cells are largely dispensable for standard nonmotor tasks. J. Neurosci. 33, 12599–12618 10.1523/JNEUROSCI.1642-13.2013 23904597 PMC6618544

[B49] Gharani N. Benayed R. Mancuso V. Brzustowicz L. M. Millonig J. H. (2004). Association of the homeobox transcription factor, ENGRAILED 2 3, with autism spectrum disorder. Mol. Psychiatry 9, 474–484 10.1038/sj.mp.4001498 15024396

[B50] Glickstein M. (2007). What does the cerebellum really do. Curr. Biol. 17, R824–R827 10.1016/j.cub.2007.08.009 17925205

[B51] Hagerman R. J. Leehey M. Heinrichs W. Tassone F. Wilson R. Hills J. (2001). Intention tremor, parkinsonism, and generalized brain atrophy in male carriers of fragile X. Neurology 57, 127–130 10.1212/WNL.57.1.127 11445641

[B52] Hall D. A. O'Keefe J. A. (2012). Fragile x-associated tremor ataxia syndrome: the expanding clinical picture, pathophysiology, epidemiology, and update on treatment. Tremor Other Hyperkinet Mov. (N.Y.) 2, 1–11 pii: tre-02-56-352-1 23439567 10.7916/D8HD7TDS PMC3570061

[B53] Hallett M. (2009). Dystonia: a sensory and motor disorder of short latency inhibition. Ann. Neurol. 66, 125–127 10.1002/ana.21762 19743461

[B54] Hamilton B. A. Frankel W. N. Kerrebrock A. W. Hawkins T. L. FitzHugh W. Kusumi K. (1996). Disruption of the nuclear hormone receptor RORalpha in staggerer mice. Nature 379, 736–739 10.1038/379736a0 8602221

[B55] Hansen S. T. Meera P. Otis T. S. Pulst S. M. (2013). Changes in Purkinje cell firing and gene expression precede behavioral pathology in a mouse model of SCA2. Hum. Mol. Genet. 22, 271–283 10.1093/hmg/dds427 23087021 PMC3526159

[B56] Hedrick A. Lee Y. Wallace G. L. Greenstein D. Clasen L. Giedd J. N. (2012). Autism risk gene MET variation and cortical thickness in typically developing children and adolescents. Autism Res. 5, 434–439 10.1002/aur.1256 23097380 PMC3528800

[B57] Hendrix C. M. Vitek J. L. (2012). Toward a network model of dystonia. Ann. N.Y. Acad. Sci. 1265, 46–55 10.1111/j.1749-6632.2012.06692.x 22823747

[B58] Hess D. T. (1982). Cerebellar nucleo-cortical neurons projecting to the vermis of lobule VII in the rat. Brain Res. 248, 361–366 10.1016/0006-8993(82)90595-9 7139282

[B59] Holmes G. (1939). The cerebellum of man. Brain 62, 2–30 10.1093/brain/62.1.113841800

[B60] Horn K. M. Pong M. Gibson A. R. (2010). Functional relations of cerebellar modules of the cat. J. Neurosci. 30, 9411–9423 10.1523/JNEUROSCI.0440-10.2010 20631170 PMC3865504

[B61] Hoshi E. Tremblay L. Feger J. Carras P. L. Strick P. L. (2005). The cerebellum communicates with the basal ganglia. Nat. Neurosci. 8, 1491–1493 10.1038/nn1544 16205719

[B62] Hourez R. Servais L. Orduz D. Gall D. Millard I. de Kerchove D'Exaerde A. (2011). Aminopyridines correct early dysfunction and delay neurodegeneration in a mouse model of spinocerebellar ataxia type 1. J. Neurosci. 31, 11795–11807 10.1523/JNEUROSCI.0905-11.2011 21849540 PMC6623197

[B63] Hutchinson M. Nakamura T. Moeller J. R. Antonini A. Belakhlef A. Dhawan V. (2000). The metabolic topography of essential blepharospasm: a focal dystonia with general implications. Neurology 55, 673–677 10.1212/WNL.55.5.673 10980732

[B64] Ichinohe N. Mori F. Shoumura K. (2000). A di-synaptic projection from the lateral cerebellar nucleus to the laterodorsal part of the striatum via the central lateral nucleus of the thalamus in the rat. Brain Res. 880, 191–197 10.1016/S0006-8993(00)02744-X 11033006

[B65] Ieraci A. Forni P. E. Ponzetto C. (2002). Viable hypomorphic signaling mutant of the Met receptor reveals a role for hepatocyte growth factor in postnatal cerebellar development. Proc. Natl. Acad. Sci. U.S.A. 99, 15200–15205 10.1073/pnas.222362099 12397180 PMC137567

[B66] Kasumu A. W. Liang X. Egorova P. Vorontsova D. Bezprozvanny I. (2012). Chronic suppression of inositol 1 4, 5-triphosphate receptor-mediated calcium signaling in cerebellar purkinje cells alleviates pathological phenotype in spinocerebellar ataxia 2 mice. J. Neurosci. 32, 12786–12796 10.1523/JNEUROSCI.1643-12.2012 22973002 PMC3470884

[B67] Klockgether T. (2010). Sporadic ataxia with adult onset: classification and diagnostic criteria. Lancet Neurol. 9, 94–104 10.1016/S1474-4422(09)70305-9 20083040

[B68] Kluge A. Kettner B. Zschenderlein R. Sandrock D. Munz D. L. Hesse S. (1998). Changes in perfusion pattern using ECD-SPECT indicate frontal lobe and cerebellar involvement in exercise-induced paroxysmal dystonia. Mov. Disord. 13, 125–134 10.1002/mds.870130124 9452337

[B69] Koekkoek S. K. Yamaguchi K. Milojkovic B. A. Dortland B. R. Ruigrok T. J. Maex R. (2005). Deletion of FMR1 in Purkinje cells enhances parallel fiber LTD, enlarges spines, and attenuates cerebellar eyelid conditioning in Fragile X syndrome. Neuron 47, 339–352 10.1016/j.neuron.2005.07.005 16055059

[B70] Kuemerle B. Gulden F. Cherosky N. Williams E. Herrup K. (2007). The mouse Engrailed genes: a window into autism. Behav. Brain Res. 176, 121–132 10.1016/j.bbr.2006.09.009 17055592 PMC2791532

[B71] LeDoux M. S. (2011). Animal models of dystonia: lessons from a mutant rat. Neurobiol. Dis. 42, 152–161 10.1016/j.nbd.2010.11.006 21081162 PMC3171987

[B70a] LeDoux M. S. Hurst D. C. Lorden J. F. (1998). Single-unit activity of cerebellar nuclear cells in the awake genetically dystonic rat. Neuroscience 86, 533–545 10.1016/S0306-4522(98)00007-4 9881867

[B70b] LeDoux M. S. Lorden J. F. (2002). Abnormal spontaneous and harmaline-stimulated Purkinje cell activity in the awake genetically dystonic rat. Exp. Brain Res. 145, 457–467 10.1007/s00221-002-1127-4 12172657

[B70c] LeDoux M. S. Lorden J. F. Ervin J. M. (1993). Cerebellectomy eliminates the motor syndrome of the genetically dystonic rat. Exp. Neurol. 120, 302–310 10.1006/exnr.1993.1064 8491286

[B72] Lemon R. N. Edgley S. A. (2010). Life without a cerebellum. Brain 133, 652–654 10.1093/brain/awq030 20305277

[B73] Liu J. Tang T. S. Tu H. Nelson O. Herndon E. Huynh D. P. (2009). Deranged calcium signaling and neurodegeneration in spinocerebellar ataxia type 2. J. Neurosci. 29, 9148–9162 10.1523/JNEUROSCI.0660-09.2009 19625506 PMC2749883

[B74] Marr D. (1969). A theory of cerebellar cortex. J. Physiol. 202, 437–470 5784296 10.1113/jphysiol.1969.sp008820 PMC1351491

[B75] Middleton F. A. Strick P. L. (2000). Basal ganglia and cerebellar loops: motor and cognitive circuits. Brain Res. Brain Res. Rev. 31, 236–250 10.1016/S0165-0173(99)00040-5 10719151

[B76] Middleton F. A. Strick P. L. (2001). Cerebellar projections to the prefrontal cortex of the primate. J. Neurosci. 21, 700–712 11160449 10.1523/JNEUROSCI.21-02-00700.2001 PMC6763818

[B77] Muller U. (2009). The monogenic primary dystonias. Brain 132, 2005–2025 10.1093/brain/awp172 19578124

[B78] Neychev V. K. Fan X. Mitev V. I. Hess E. J. Jinnah H. A. (2008). The basal ganglia and cerebellum interact in the expression of dystonic movement. Brain 131, 2499–2509 10.1093/brain/awn168 18669484 PMC2724906

[B79] Neychev V. K. Gross R. E. Lehericy S. Hess E. J. Jinnah H. A. (2011). The functional neuroanatomy of dystonia. Neurobiol. Dis. 42, 185–201 10.1016/j.nbd.2011.01.026 21303695 PMC3478782

[B80] Niethammer M. Carbon M. Argyelan M. Eidelberg D. (2011). Hereditary dystonia as a neurodevelopmental circuit disorder: evidence from neuroimaging. Neurobiol. Dis. 42, 202–209 10.1016/j.nbd.2010.10.010 20965251 PMC3062649

[B81] Odergren T. Stone-Elander S. Ingvar M. (1998). Cerebral and cerebellar activation in correlation to the action-induced dystonia in writer's cramp. Mov. Disord. 13, 497–508 10.1002/mds.870130321 9613744

[B82] Ozol K. Hayden J. M. Oberdick J. Hawkes R. (1999). Transverse zones in the vermis of the mouse cerebellum. J. Comp. Neurol. 412, 95–111 10.1002/(SICI)1096-9861(19990913)412:1<95::AID-CNE7>3.3.CO;2-P 10440712

[B83] Palkovits M. Magyar P. Szentagothai J. (1972). Quantitative histological analysis of the cerebellar cortex in the cat. IV. Mossy fiber-Purkinje cell numerical transfer. Brain Res. 45, 15–29 10.1016/0006-8993(72)90213-2 4116421

[B84a] Pizoli C. E. Jinnah H. A. Billingsley M. L. Hess E. J. (2002). Abnormal cerebellar signaling induces dystonia in mice. J. Neurosci. 22, 7825–7833 12196606 10.1523/JNEUROSCI.22-17-07825.2002 PMC6757989

[B84] Preibisch C. Berg D. Hofmann E. Solymosi L. Naumann M. (2001). Cerebral activation patterns in patients with writer's cramp: a functional magnetic resonance imaging study. J. Neurol. 248, 10–17 10.1007/s004150170263 11266013

[B85] Prudente C. N. Pardo C. A. Xiao J. Hanfelt J. Hess E. J. Ledoux M. S. (2013). Neuropathology of cervical dystonia. Exp. Neurol. 241, 95–104 10.1016/j.expneurol.2012.11.019 23195594 PMC3570661

[B86] Raike R. S. Pizoli C. E. Weisz C. van den Maagdenberg A. M. Jinnah H. A. Hess E. J. (2012). Limited regional cerebellar dysfunction induces focal dystonia in mice. Neurobiol. Dis. 49C, 200–210 10.1016/j.nbd.2012.07.019 22850483 PMC3567246

[B87] Raike R. S. Weisz C. Hoebeek F. E. Terzi M. C. De Zeeuw C. I. van den Maagdenberg A. M. (2013). Stress, caffeine and ethanol trigger transient neurological dysfunction through shared mechanisms in a mouse calcium channelopathy. Neurobiol. Dis. 50, 151–159 10.1016/j.nbd.2012.09.005 23009754 PMC3534906

[B88] Reeber S. L. White J. J. George-Jones N. A. Sillitoe R. V. (2012). Architecture and development of olivocerebellar circuit topography. Front. Neural Circuits 6:115 10.3389/fncir.2012.00115 23293588 PMC3534185

[B89] Reith R. M. McKenna J. Wu H. Hashmi S. S. Cho S. H. Dash P. K. (2013). Loss of Tsc2 in Purkinje cells is associated with autistic-like behavior in a mouse model of tuberous sclerosis complex. Neurobiol. Dis. 51, 93–103 10.1016/j.nbd.2012.10.014 23123587

[B90] Ritvo E. R. Freeman B. J. Scheibel A. B. Duong T. Robinson H. Guthrie D. (1986). Lower Purkinje cell counts in the cerebella of four autistic subjects: initial findings of the UCLA-NSAC Autopsy Research Report. Am. J. Psychiatry 143, 862–866 3717426 10.1176/ajp.143.7.862

[B91] Ruigrok T. J. (2011). Ins and outs of cerebellar modules. Cerebellum 10, 464–474 10.1007/s12311-010-0164-y 20232190 PMC3169761

[B92] Sarna J. R. Hawkes R. (2003). Patterned Purkinje cell death in the cerebellum. Prog. Neurobiol. 70, 473–507 10.1016/S0301-0082(03)00114-X 14568361

[B93] Schmahmann J. D. (2010). The role of the cerebellum in cognition and emotion: personal reflections since 1982 on the dysmetria of thought hypothesis, and its historical evolution from theory to therapy. Neuropsychol. Rev. 20, 236–260 10.1007/s11065-010-9142-x 20821056

[B94] Schorge S. van de Leemput J. Singleton A. Houlden H. Hardy J. (2010). Human ataxias: a genetic dissection of inositol triphosphate receptor (ITPR1)-dependent signaling. Trends Neurosci. 33, 211–219 10.1016/j.tins.2010.02.005 20226542 PMC4684264

[B95] Scott J. A. Schumann C. M. Goodlin-Jones B. L. Amaral D. G. (2009). A comprehensive volumetric analysis of the cerebellum in children and adolescents with autism spectrum disorder. Autism Res. 2, 246–257 10.1002/aur.97 19885834 PMC2999464

[B96] Sen B. Singh A. S. Sinha S. Chatterjee A. Ahmed S. Ghosh S. (2010). Family-based studies indicate association of Engrailed 2 gene with autism in an Indian population. Genes Brain Behav. 9, 248–255 10.1111/j.1601-183X.2009.00556.x 20050924

[B97] Serra H. G. Duvick L. Zu T. Carlson K. Stevens S. Jorgensen N. (2006). RORalpha-mediated Purkinje cell development determines disease severity in adult SCA1 mice. Cell 127, 697–708 10.1016/j.cell.2006.09.036 17110330

[B98] Sgaier S. K. Lao Z. Villanueva M. P. Berenshteyn F. Stephen D. Turnbull R. K. (2007). Genetic subdivision of the tectum and cerebellum into functionally related regions based on differential sensitivity to engrailed proteins. Development 134, 2325–2335 10.1242/dev.000620 17537797 PMC2840613

[B99] Shakkottai V. G. do Carmo Costa M. Dell'Orco J. M. Sankaranarayanan A. Wulff H. Paulson H. L. (2011). Early changes in cerebellar physiology accompany motor dysfunction in the polyglutamine disease spinocerebellar ataxia type 3. J. Neurosci. 31, 13002–13014 10.1523/JNEUROSCI.2789-11.2011 21900579 PMC3170039

[B100] Shamim E. A. Chu J. Scheider L. H. Savitt J. Jinnah H. A. Hallett M. (2011). Extreme task specificity in writer's cramp. Mov. Disord. 26, 2107–2109 10.1002/mds.23827 21714006 PMC3417074

[B101] Sillitoe R. V. Marzban H. Larouche M. Zahedi S. Affanni J. Hawkes R. (2005). Conservation of the architecture of the anterior lobe vermis of the cerebellum across mammalian species. Prog. Brain Res. 148, 283–297 10.1016/S0079-6123(04)48022-4 15661197

[B102] Smit A. E. van der Geest J. N. Vellema M. Koekkoek S. K. Willemsen R. Govaerts L. C. (2008). Savings and extinction of conditioned eyeblink responses in fragile X syndrome. Genes Brain Behav. 7, 770–777 10.1111/j.1601-183X.2008.00417.x 18616611 PMC2613242

[B103] Stanfield A. C. McIntosh A. M. Spencer M. D. Philip R. Gaur S. Lawrie S. M. (2008). Towards a neuroanatomy of autism: a systematic review and meta-analysis of structural magnetic resonance imaging studies. Eur. Psychiatry 23, 289–299 10.1016/j.eurpsy.2007.05.006 17765485

[B104] Stoodley C. J. Schmahmann J. D. (2009). Functional topography in the human cerebellum: a meta-analysis of neuroimaging studies. Neuroimage 44, 489–501 10.1016/j.neuroimage.2008.08.039 18835452

[B105] Stoodley C. J. Schmahmann J. D. (2010). Evidence for topographic organization in the cerebellum of motor control versus cognitive and affective processing. Cortex 46, 831–844 10.1016/j.cortex.2009.11.008 20152963 PMC2873095

[B106a] Teo J. T. van de Warrenburg B. P. Schneider S. A. Rothwell J. C. Bhatia K. P. (2009). Neurophysiological evidence for cerebellar dysfunction in primary focal dystonia. J. Neurol. Neurosurg. Psychiatry. 80, 80–83 10.1136/jnnp.2008.144626 19091711

[B106] Tobia M. J. Woodruff-Pak D. S. (2009). Delay eyeblink classical conditioning is impaired in Fragile X syndrome. Behav. Neurosci. 123, 665–676 10.1037/a0015662 19485573 PMC2814536

[B107] Tolbert D. L. Bantli H. Bloedel J. R. (1976). Anatomical and physiological evidence for a cerebellar nucleo-cortical projection in the cat. Neuroscience 1, 205–217 10.1016/0306-4522(76)90078-6 11370232

[B108] Tolbert D. L. Ewald M. Gutting J. La Regina M. C. (1995). Spatial and temporal pattern of Purkinje cell degeneration in shaker mutant rats with hereditary cerebellar ataxia. J. Comp. Neurol. 355, 490–507 10.1002/cne.903550403 7636028

[B109] Tsai P. T. Hull C. Chu Y. Greene-Colozzi E. Sadowski A. R. Leech J. M. (2012). Autistic-like behaviour and cerebellar dysfunction in Purkinje cell Tsc1 mutant mice. Nature 488, 647–651 10.1038/nature11310 22763451 PMC3615424

[B110] Ulug A. M. Vo A. Argyelan M. Tanabe L. Schiffer W. K. Dewey S. (2011). Cerebellothalamocortical pathway abnormalities in torsinA DYT1 knock-in mice. Proc. Natl. Acad. Sci. U.S.A. 108, 6638–6643 10.1073/pnas.1016445108 21464304 PMC3081027

[B111] Uusisaari M. De Schutter E. (2011). The mysterious microcircuitry of the cerebellar nuclei. J. Physiol. 589, 3441–3457 10.1113/jphysiol.2010.201582 21521761 PMC3167109

[B112] Wadiche J. I. Jahr C. E. (2005). Patterned expression of Purkinje cell glutamate transporters controls synaptic plasticity. Nat. Neurosci. 8, 1329–1334 10.1038/nn1539 16136036

[B113] Walter J. T. Alvina K. Womack M. D. Chevez C. Khodakhah K. (2006). Decreases in the precision of Purkinje cell pacemaking cause cerebellar dysfunction and ataxia. Nat. Neurosci. 9, 389–397 10.1038/nn1648 16474392

[B114] Wang L. Jia M. Yue W. Tang F. Qu M. Ruan Y. (2008). Association of the ENGRAILED 2 (EN2) gene with autism in Chinese Han population. Am. J. Med.l Genet. B Neuropsychiatr. Genet. 147B, 434–438 10.1002/ajmg.b.30623 17948901

[B115] Welsh J. P. Yuen G. Placantonakis D. G. Vu T. Q. Haiss F. O'Hearn E. (2002). Why do Purkinje cells die so easily after global brain ischemia? Aldolase, C, EAAT4, and the cerebellar contribution to posthypoxic myoclonus. Adv. Neurol. 89, 331–359 11968459

[B116] White J. Sillitoe R. V. (2013). Development of the cerebellum: from gene expression patterns to circuit maps. WIREs Dev. Biol. 2, 149–164 10.1002/wdev.65 23799634

[B117] Whitney E. R. Kemper T. L. Bauman M. L. Rosene D. L. Blatt G. J. (2008). Cerebellar Purkinje cells are reduced in a subpopulation of autistic brains: a stereological experiment using calbindin-D28k. Cerebellum 7, 406–416 10.1007/s12311-008-0043-y 18587625

[B118] Yang P. Shu B. C. Hallmayer J. F. Lung F. W. (2010). Intronic single nucleotide polymorphisms of engrailed homeobox 2 modulate the disease vulnerability of autism in a han chinese population. Neuropsychobiology 62, 104–115 10.1159/000315441 20523082

[B119a] Yokoi F. Dang M. T. Li Y. (2012). Improved motor performance in Dyt1 ΔGAG heterozygous knock-in mice by cerebellar Purkinje-cell specific Dyt1 conditional knocking-out. Behav. Brain Res. 230, 389–398 10.1016/j.bbr.2012.02.029 22391119 PMC3322286

[B119] Zhao Y. Sharma N. LeDoux M. S. (2011). The DYT1 carrier state increases energy demand in the olivocerebellar network. Neuroscience 177, 183–194 10.1016/j.neuroscience.2011.01.015 21241782 PMC3171990

